# Prevention Better Than Cure

**Published:** 1885-12

**Authors:** 


### PREVENTION BETTER THAN CURE.

In a recent book by Marion Harland, "Common Sense in the

Nursery," she attributes much of the babies' sufferings from cold to the placing of the children on the floor to play. She says: "In the best warmed room there is inevitably a current of cooled air close to the floor, in which as baby sits on the carpet his feet are bathed, while his shoulders may over-top it." She considers another prolific source of holding of the young monarch close to the window, the air in close proximity to the window-panes being several degrees cooler than that further in the room, and more or less draughts through the caseings of the window can not be prevented.

Sudden changes from the room used as a nursery to the halls, or rooms with lower temperature, are another evil, which might be avoided by extra wraps when it is necessary to expose a baby to such a change. Flannel night-dresses coming below the feet far enough to allow of being drawn tightly at the hem, forming a bag, without preventing the baby using his feet with perfect freedom, are one of the means to prevent exposure at night. Marion Harland suggests loops on the lower edges of the mattress, and corresponding buttons on the blankets, as a safeguard against exposure to cold in the night.

In the daytime have a mattress covered to put on the floor for baby to stretch and roll on, or a box with sides not more than five or six inches high, entirely padded on the inside, and large enough to the baby and his toys; this, with a baby-chair having a table at-hold tachment will prevent hours of suffering, anxiety, and toil.

One wise mother, whose babies are the pictures of health, bundles them up every day as warmly as though they were to go into the outer air, and they are carried, and allowed to run when large enough, about a room the windows of which are all open. This allows them to have all the benefit possible from outer air when the weather is so inclement that they could not be taken out of doors. These babies rarely have a cold, and when met out of doors in winter they are a charming sight. Apparently they are clad in such a way as to be perfectly proof against cold. Soft woolen dresses and cloaks, hood tied closely under the chin warm leggins, thick-soled shoes without heels, and, when the snow is upon the ground, rubber boots complete their costume. With red cheeks, shining eyes and clear, ringing voices, they are the embodiment of healthy and childhood.

Tiredness has a variety of ways of manifesting itself. At the time of the fire in the Hudson tannery, a well known citizen of that burg fought the fire with water and something else to keep it from striking in, and when he went home he was so tired that—well, the way his daughter expressed it was, "pa was so tired that he staggered."—*The Marlboro Times.*

